# Shotgun proteomics of the barley seed proteome

**DOI:** 10.1186/s12864-016-3408-5

**Published:** 2017-01-06

**Authors:** Ramamurthy Mahalingam

**Affiliations:** USDA, Agricultural Research Service, Cereal Crops Research Unit, 502 Walnut Street, Madison, WI 53726 USA

**Keywords:** Barley, Gene ontologies, GO enrichment, Hordoindolines, Hydropathicity, Mass spectrometry, Nano liquid chromatography, Proteome, Seed, Six-rowed, Spectral counting, Two-rowed

## Abstract

**Background:**

Barley seed proteins are of prime importance to the brewing industry, human and animal nutrition and in plant breeding for cultivar identification. To obtain comprehensive proteomic data from seeds, total protein from a two-rowed (Conrad) and a six-rowed (Lacey) barley cultivar were precipitated in acetone, digested *in-solution*, and the resulting peptides were analyzed by nano-liquid chromatography coupled with tandem mass spectrometry.

**Results:**

The raw mass spectra data searched against Uniprot’s Barley database using in-house *Mascot* search engine identified 1168 unique proteins. Gene Ontology (GO) analysis indicated that the majority of the seed proteins were cytosolic, with catalytic activity and associated with carbohydrate metabolism. Spectral counting analysis showed that there are 20 differentially abundant seed proteins between the two-rowed Conrad and six-rowed Lacey cultivars.

**Conclusion:**

This study paves the way for the use of a top-down gel-free proteomics strategy in barley for investigating more complex traits such as malting quality. Differential abundance of hordoindoline proteins impact the seed hardness trait of barley cultivars.

**Electronic supplementary material:**

The online version of this article (doi:10.1186/s12864-016-3408-5) contains supplementary material, which is available to authorized users.

## Background

In terms of tonnage, world-wide production of barley ranks fourth among cultivated cereals. More than 60% of the barley produced is used by the brewing industry. Barley seed germination is the foundation of malting and brewing industry. Hence it is not surprising that barley has evolved as a model for seed germination research. The total protein content in barley seed varies between 8 and 15% [1]. The amount and composition of barley proteins influence the suitability and quality of grain for its end uses, with approximately a third of the proteins being present in the final beer [2].

Hordeins, the storage proteins in barley, account for nearly 80% of the total proteins [3]. Two- dimension gel electrophoresis (2-DE) was used to separate barley seed proteins [4–8]. Seed tissue sub-proteomes including plasma membrane, endosperm, embryo, and aleurone layer have been analyzed using 2-DE combined with mass spectrometry which led to the identification of hundreds of proteins [9]. Some of the recent advances in the proteomics field such as shotgun proteomics have not been explored in barley. In shot gun proteomics (bottom-up strategy), complex peptide fractions generated after protein proteolytic digestion can be resolved using different fractionation strategies, which offer high-throughput analyses of the proteome of an organ, organelle or a cell type, and provide a snapshot of the major protein constituents [10]. One of the recent trends in shotgun proteomics is the use of label-free methods for protein quantitation [11]. A number of reports on the use of gel-free label-free quantitative proteomics have been conducted in plants including Arabidopsis [12], tomato [13], soybeans [14], barley [15] and corn [16].

Wild barley, *Hordeum vulgare* ssp. *spontaneum*, the progenitor of cultivated barley has two-rows of seeds (kernels) in each head (spike). A single recessive gene, *vrs1,* has been shown to cause the six-row phenotype [17]. Morphologically, two-row barley kernels tend to be symmetrical, while six-row barley has symmetrical center but lateral rows are shorter, thinner and slightly twisted (Additional file 1). Intuitively, a six-rowed spike can stably produce three times the usual grain number compared to a two-rowed type and hence may have been selected by plant breeders. From a brewers’ view-point, six-row barley may be less desirable compared to a two-row owing to non-uniformity of the seed size of the former. Furthermore, six-row barley tend to have more protein content and hence less starch than the latter [18]. Through rigorous breeding efforts a number of two-row and six-row barley cultivars with desirable malting quality and disease resistance traits have been commercialized. However, the differences in the protein constituents between six-row and two-row barley seeds have not been investigated. In this study a shot gun proteomics strategy was employed in order to provide a deeper characterization of the barley seed proteome. Spectral counting analysis was undertaken to identify differentially abundant proteins in the seeds of two-rowed Conrad and six-rowed Lacey barley cultivars.

## Results and discussion

Mature dry seeds of barley are the primary raw materials for the malting and brewing industry. In this study we undertook a deep proteome analysis of the fully matured dry seeds of the two-row, Conrad and six-row, Lacey cultivar. Averaging the triplicate peptide profiles from these two lines generated 71,464 spectra that could be mapped to 1185 proteins with unique Uniprot identifiers. Eleven of these protein sequences showed matches to the decoy database. This indicates that the false discovery rate in the current study is 0.93%. Six proteins that were identified as keratin (5) or trypsin (1) were removed from the analysis, thus giving a set of 1168 proteins for further detailed analysis. Using a similar nanoLC MS/MS strategy for seed proteome analysis, 243 non-redundant proteins were reported for soybeans [19] and 352 for quinoa [20]. This 3–4 fold higher number of seed proteins identified in our analysis indicates that the seed protein extraction, digestion and nanoLC MS/MS analysis were superior to those reported for soybeans [17] and quinoa [18]. In one of the most comprehensive proteome exploration studies using multidimensional protein identification technology (Mudpit), 822 seed proteins were reported in rice [21]. Recently, deep proteome analysis of the gerantoplasts from the inner integuments of the developing seeds of *Jatropha curcas* using an in-solution digestion followed by LC MS/MS identified 812 proteins [22]. A comparison of the seed proteomes of the various *opaque* mutants of maize identified nearly 2700 proteins using the LC MS/MS strategy [16]. Thus the number of proteins identified in the current study is comparable to other deep proteome studies in the recently published literature.

Protein profiling studies in barley were conducted even before the inception of the concept of proteomics [9]. Nearly 10 different studies have been reported on barley seed proteome analysis using the 2DE coupled with the MALDI-TOF peptide mass fingerprinting and/or mass spectrometry. Information provided in these aforementioned studies, especially protein descriptions, molecular weight and isoelectric point (pI), were used to compare with the results from the current study (Table 1). Nearly 85% (220) of the proteins reported in the earlier studies (259) were identified in this analysis. A comparison between the 2DE and a gel-free MudPit analysis in rice indicated that about 29% of the proteins identified were unique to the former, suggesting that inclusion of two different techniques can be complementary and provide a more comprehensive proteome coverage [21]. The comparative analysis undertaken here indicates 15% of the proteins were unique to the 2DE technique and begs the question of identity of those proteins. An obvious case in point relates to the study of barley peroxidases [21] (Table 1). The three reported peroxidases in the European cultivar Sloop were not present in the two American cultivars used in this study. In the current study six different peroxidases were identified, but based on their theoretical pI and MW none of them seem to be close to those reported earlier [23]. Thus some of the proteins may be unique to the cultivars investigated. Other commonly missing proteins in the current study compared with studies summarized in Table 1 included barwin, small heat shock proteins, cold regulated protein, and isoflavone reductase. These stress response proteins may be influenced by the environment in which the plants were grown and conditions during seed set.

**Table 1:** Overlap between protein identification from other barley seed proteome studies compared with the current study

Seed tissue	Cultivar(s)	Unique proteins identified	Overlap with current study	Reference
Whole seeds	Barke	27	25	[49]
Whole seeds	Barke	103	88	[26]
Whole seeds	Barke, Golden Promise	5	5	[50]
Whole seeds	Multiple cvs	14	12	[9]
Whole seeds	Sloop	3	0	[23]
Whole seeds	DOM, REC	20	19	[51]
Whole seeds	Esterel	23	20	[52]
Aleurone	Himalaya	36	28	[24]
Endosperm	Barke	9	8	[53]
Aleurone, embryo, endopserm	Barke	19	15	[54]

In one of the earlier seed proteome studies, plasma membrane proteins from barley aleurone were enriched using reverse-phase chromatography, SDS-PAGE and LC-MS/MS [24]. Of the 36 proteins with trans-membrane (TM) domains, 28 were identified in our analysis. Using the barley uniprot identifiers, the information for TM domain (number of domains and their co-ordinates) was retrieved from the UniportKB database and identified 74 proteins with one or more TM domains (Additional file 2). This suggests that the methodology used for the protein extraction in the current study is compatible even for the more tenacious membrane proteins.

**Fig. 1:**
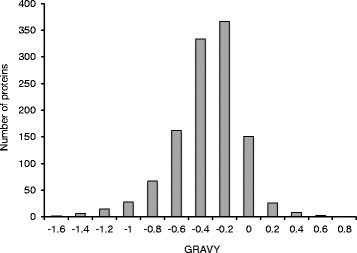
Distribution of barley seed proteins based on their hydropathicity. Full-length protein sequences were used to calculate the Grand Average of Hydropathicity (GRAVY). Negative values indicate hydrophilic proteins and positive values indicate hydrophobic proteins. Histogram was generated using MS Excel

The grand average of hydropathicity (GRAVY) index for the 1168 proteins identified in this study was compared using the histogram function in Excel (Fig. 1). Proteins with negative GRAVY scores are hydrophilic and those with positive values are hydrophobic. The majority of proteins had a GRAVY score ranging between −0.8 and 0, indicating that most of them are hydrophilic. The asymmetric distribution of the GRAVY values (Skewness: −0.58 and Kurtosis =1.84) confirmed the left-heavy tails of the distribution. A similar distribution of the proteins in rice seeds was reported [25]. The tendency of the barley seed proteome for hydrophilicity suggests that these water soluble proteins may be active in physiological processes during imbibition and subsequently during germination.

**Fig. 2:**
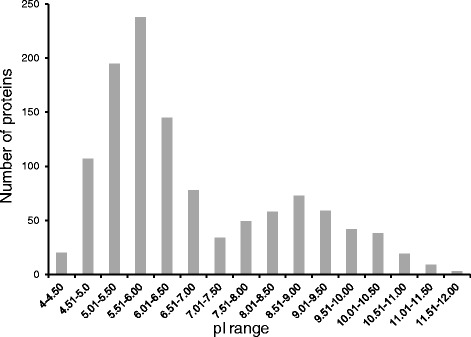
Distribution of barley seed proteins based on their isolectric points. Theoretical pI values of the proteins were obtained from the Uniprot database. The pI values were binned into 0.5 units and histogram was generated using MS Excel

Traditional proteomics strategies such as 2DE are conducted to examine particular groups of proteins based on their solubility or pI etc. For example, soluble seed proteins were extracted using a weak buffer at neutral pH since many of the well-studied seed proteins (e.g. amylases, subtilisin inhibitors, chitinases, non-specific lipid transfer proteins) were isolated under these conditions and minimized the extraction of seed storage proteins that would otherwise dominate the 2-DE profile and mask the lower abundance proteins [9]. The use of extraction buffer containing Tris–HCl and KCl in the current study was not favorable for solubilizing the abundant seed storage proteins like hordeins. This in turn favored the identification of lower abundance proteins. Another strategy for proteome analysis was to separate proteins by focusing them for a defined pI range [24, 26]. Using the top-down proteomics strategy described here, the theoretical pI values of the 1168 proteins ranged from 4–12 (Fig. 2). The pI value distribution showed a bi-modal pattern with the majority of the seed proteins in the 4–7 range. Nearly 250 proteins were in the 5.5–6 pI range. A second peak was observed in the alkaline pI range with more than 50 proteins with a pI of 8.5–9. This unbiased technique (w.r.t pI) thus enabled a deeper analysis of the seed proteome.

For the 1168 unique proteins of barley in the UniprotKB database, meaningful annotations were available for only about 241 proteins (21%). Uncharacterized proteins comprised about 60% (707) of the seed proteome while the remaining 19% (220) of the proteome comprised of predicted proteins. To improve the annotations, barley Uniref identifiers were mapped to the Uniref90 and Uniref50 data sets. The 1168 barley Uniprot identifiers mapped to 1094 entries from the Uniref90 database and 813 entries in the Uniref50 database. Using the mapping information to the Uniref90 and Uniref50 databases, we manually added descriptions for nearly 80 proteins (Additional file 3).

**Fig. 3:**
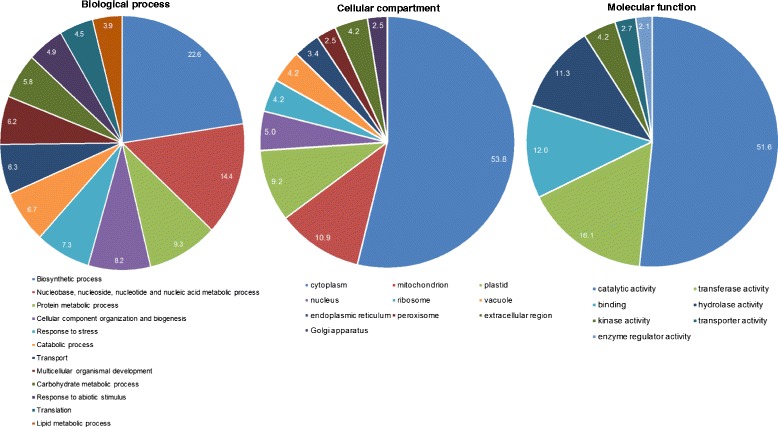
Pie charts of Gene Ontologies (GO) of the barley seed proteins. For each of the GO categories only terms with more than 2% of the total were included for this analysis. The numbers on the chart represent the percentage of proteins in each GO category

Identified seed proteins were classified by Gene Ontology (GO) terms in three broad domains – biological process, cellular component and molecular function. About 1060 proteins were associated with one or more GO terms, while 108 proteins did not have any GO annotations. In the molecular function category, 891 proteins were associated with 1370 GOs. In the biological process category, 697 proteins were associated with 1421 GO terms, and the cellular compartment or localization category, 569 proteins were associated with 852 GO terms. The large number of GO terms is attributed to the differences in the amount of information available for some of the well characterized proteins with detailed annotations. A careful analysis of the GO terms showed that the number of unique GO identifiers were 468, 357 and 107 for the domains of biological process, molecular function and cellular compartment, respectively. To further reduce this complexity and provide an easy visual of the major GO terms associated with the seed proteome, the CateGOrizer program was used [27]. In conjunction with the plant GO slim terms as the background, this analysis indicated that there were 41, 21, and 27 GO terms associated with the biological process, molecular function and cellular compartment, respectively (Fig. 3). Nearly a quarter of the proteome was associated with metabolic processes (nucleic acids, proteins, lipids, carbohydrate metabolism), 18% of the proteins were associated with biosynthetic processes and about 12% were related to proteins responsive to stress. While proteins associated with translation were identified in the seed proteome, we did not identify many proteins associated with transcriptional machinery. This is consistent with earlier reports that the dry seeds accumulate translatable RNA (i.e., stored mRNA) that is produced during seed development [28] and that *de novo* transcription is not essential for early stages of seed germination [29].

### GO enrichment analysis

Identifying enriched GOs among the seed proteins aids in determining key biological processes, vital molecular functions and organelles within seeds in which these proteins localize. Since detailed annotations for many of the genes in the barley genome were not available, rice orthologs of the barley seed proteins were identified. A total of 1166 rice proteins matching barley (E-value > 1e10^−5^ and with at least 100 HSPs) were retrieved by BLAST analysis. Among these, 874 unique TIGR gene identifiers were retrieved and these proteins had detailed GO annotations. These unique rice proteins were subjected to singular enrichment analysis (SEA) in agriGO to identify enriched GOs [30]. This analysis is designed to identify enriched GO terms in a list of probe sets or gene identifiers. Finding enriched GO terms corresponds to finding enriched biological facts, and term enrichment level is judged by comparing the query list to a background population (54,971 *Oryza sativa Japonica* proteins, MSU6.1 version) from which the query list is derived. A total of 68 enriched GO terms were identified, of which 27 were associated with biological processes, 15 with molecular function and 26 with cellular component (Additional file 4).

**Fig. 4:**
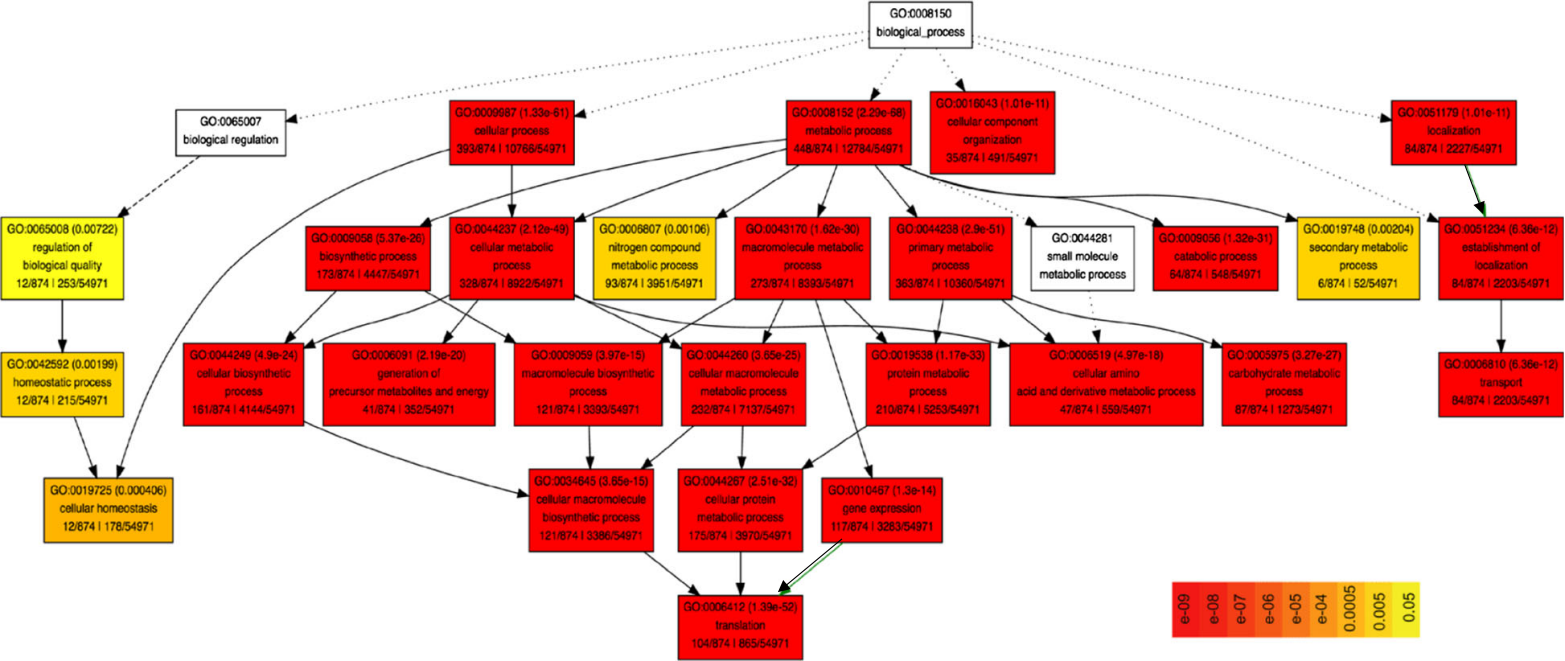
Gene Ontology enrichment analysis of barley seed proteins using AgriGO. Each box shows the GO term number, the *p*-value in parenthesis, GO term. The first pair of numerals represents the number of proteins in the input list associated with that GO term and the number of proteins in the input list. The second pair of numerals represents the number of proteins associated with the particular GO term in the rice database and the total number of rice proteins with GO annotations in the rice database. The box colors indicate levels of statistical significance with yellow = 0.05; orange = e-05 and red = e-09. Dotted arrows indicate two or more significant nodes, and dashed arrows indicate one significant node

Consistent with the GO analysis, proteins associated with metabolism were enriched and 87 proteins in particular associated with carbohydrate metabolism (Fig. 4). Among the 47 proteins associated with the amino acid metabolic process, 19 (40%) of them were involved in various amino acid biosynthetic pathways and the remainder 28 were proteins associated with aminoacyl tRNA synthase activity. All the 12 proteins associated with cellular homeostasis were in fact important in redox regulation, further supporting the recent findings about the role of reactive oxygen species in seed dormancy and germination [31, 32]. More than 100 proteins were associated with translation and nearly 60% of these proteins were structural components of the ribosome machinery. One of the interesting enriched GO terms was transport that included 84 proteins involved in intracellular trafficking, signal recognition particle, transport of metal ions, lipids, and nutrients. Among the 41 proteins involved in the generation of precursor metabolites and energy, the majority of them were associated with glycolysis, tricaboxylic acid cycle or gluconeogenesis.

The enriched GO terms associated with molecular function were considerably fewer compared with the biological processes (Additional file 5). Of the 48 proteins associated with nucleoside-triphosphatase activity, 21 proteins had GTPase activity. Among the 86 proteins with transferase activity, 30 proteins were kinases suggesting that phosphorylation of seed proteins may play an important role during the transition from quiescence to imbibition and germination in barley. The importance of phosphorylation during seed imbibition and germination has been demonstrated in maize [33], rice [34] and oak [35]. The three major steps of protein synthesis namely – initiation, elongation and termination were represented in the seed proteome. Of the 18 proteins associated with translation factor activity, nine were associated with initiation, eight proteins were elongation factors, while one protein had translation termination activity.

The cellular component GO enrichment terms were consistent with the major GO categories that were identified using the barley identifiers (Additional file 6). The largest number of proteins were localized to the cytoplasm (179) while nuclear proteins were not significantly enriched in the seed proteome. This again indicates that the vast majority of the seed proteome consists of soluble proteins consistent with the hydropathicity profile described earlier. Interestingly, the second largest group of 85 proteins were associated with plasma membrane, and may be involved in the process of protein mobilization during germination [36]. The third largest group of 71 proteins were associated with ribosomes, further confirming the importance of protein translation in seeds.

### Differences in two-row versus six-row barley seed proteome

Spectral counting is based on the rationale that peptides from more abundant proteins will be selected more frequently for fragmentation and will thus produce a higher number of MS/MS spectra. Thus, the number of MS/MS scans is tabulated, and the protein abundance is inferred from the total number of MS/MS spectra that match peptides from the protein [37]. Spectral counting is becoming popular in label-free quantification due to its simple procedure that does not require chromatographic peak integration or retention time alignment [10].

In this study we examined the differentially abundant proteins in the two-rowed Conrad when compared with the six-rowed Lacey seed samples. Differential expression was based on statistical significance of the averaged differences in the spectral counts between the two cultivars (Additional files 7 and 8). It should be noted that the overall seed protein profiles as observed on a one-dimensional SDS-PAGE was similar for the two cultivars (Additional file 9). Of the 1168 proteins, 20 proteins differed in their abundances between the two cultivars (Table 2). Eleven of these proteins were in higher abundance in Lacey and nine of them in Conrad. It is interesting to note that two different sucrose synthase proteins showed opposite patterns of abundance in the two cultivars. The gene encoding the larger proteins SS1 is localized to chromosome 7, and the gene for the homologous shorter version, SS2, is on chromosome 2 [38]. Both of these proteins are more abundant in the endosperm tissues than in aleurone layer [39]. However, the biological significance of their differential abundance in the two-rowed Conrad versus the six-rowed Lacey is not clear.

**Table 2:** Differentially abundant proteins between two-rowed Conrad and six-rowed Lacey cultivars based on spectral counting analysis

			Probability% (Number of peptides)					
Description	ID	*P*-value	Conrad_1	Conrad_2	Conrad_3	Lacey_1	Lacey_2	Lacey_3
Uncharacterized protein; putative gliadin	M0XYT2	0.00043	100% (37)	100% (36)	100% (40)	100% (70)	100% (45)	100% (54)
Uncharacterized protein; putative ser-type endopeptidase inhibitor	M0Y075	0.00039	100% (17)	100% (19)	100% (20)	100% (29)	100% (31)	100% (38)
Lipoxygenase	M0WRG0	<0.00010	100% (36)	100% (33)	100% (37)	100% (55)	100% (57)	100% (57)
Putative aldehyde dehydrogenase	M0UEJ7	<0.00010	93% (1)	44% (0)	23% (0)	100% (5)	100% (5)	100% (7)
Uncharacterized protein; putative hydrolase	M0YBZ9	<0.00010	100% (6)	100% (5)	100% (6)	100% (15)	100% (18)	100% (16)
Malic enzyme	F2ELT5	<0.00010	100% (29)	100% (29)	100% (29)	100% (48)	100% (53)	100% (48)
Uncharacterized protein; putative globulin	M0XH58	<0.00010	100% (268)	100% (292)	100% (335)	100% (307)	100% (362)	100% (404)
Sucrose synthase	M0UKI5	<0.00010	100% (208)	100% (165)	100% (166)	100% (252)	100% (231)	100% (217)
Hordoindoline b-1	Q5URW6	<0.00010	100% (36)	100% (21)	100% (27)	100% (66)	100% (47)	100% (44)
Uncharacterized protein; putative globulin	M0XH59	<0.00010	100% (139)	100% (136)	100% (157)	100% (204)	100% (199)	100% (207)
Uncharacterized protein	M0XUU4	<0.00010	100% (0)	100% (40)	100% (79)	100% (157)	100% (193)	100% (183)
Uncharacterized membrane protein	M0Z4S0	0.00024	100% (108)	100% (89)	100% (93)	100% (75)	100% (62)	100% (73)
Uncharacterized vacuolar sorting receptor	M0UNJ0	0.00014	100% (4)	100% (8)	100% (4)	57% (1)	0% (0)	0% (0)
Putative sHSP	M0Z6T1	0.00014	100% (22)	100% (28)	100% (28)	100% (13)	100% (13)	100% (12)
Uncharacterized Alpha-amylase inhibitor BMAI-1	M0UYA9	0.00014	100% (81)	100% (62)	100% (58)	100% (42)	100% (41)	100% (50)
Sucrose synthase	M0XEF6	<0.00010	100% (142)	100% (96)	100% (91)	100% (85)	100% (74)	100% (67)
Putative aspartic-type endopeptidase	M0W9B2	<0.00010	100% (15)	100% (14)	100% (16)	100% (4)	100% (5)	100% (2)
Uncharacterized protein ; putative sHSP	M0YEG9	<0.00010	100% (21)	100% (21)	100% (20)	0% (0)	0% (0)	60% (16)
Hordoindoline a	Q5URW5	<0.00010	100% (32)	100% (25)	100% (26)	100% (4)	100% (8)	100% (6)
Hordoindoline b-2	Q5URW7	<0.00010	100% (73)	100% (71)	100% (72)	100% (7)	100% (6)	99% (2)

**Fig. 5:**
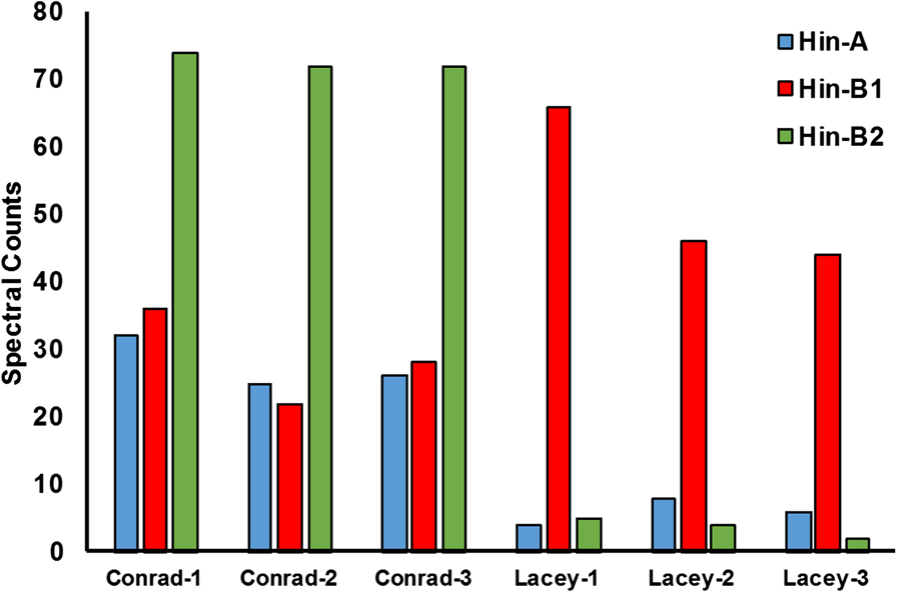
Spectral count analysis of the barley hardoindoline proteins in the seeds of two-rowed Conrad and six-rowed Lacey cultivar. The number of spectra for Hin-A, Hin-B1 and Hin-B2 for Conrad and Lacey cultivars from three biological replicates are shown here

It was reported that milling energy, another measure of grain hardness, correlates negatively with malting quality in barley [40]. Therefore, the development of softer cultivars may benefit malting quality traits. Hordoindolines are proteins homologous to the puroindolines of wheat, which are important for determining the grain hardiness [41–43] and endosperm texture [44]. In barley there are three hordoindolines – Hin-A, Hin-B1 and Hin-B2 [45]. In this study we found a significantly higher level of Hin-A and Hin-B2 in Conrad, while the levels of Hin-B1 were higher in Lacey (Fig. 5). On the contrary, Hin-A and Hin-B1 protein abundances did not vary in two-rowed Shikaku hakada and six-rowed Ichibanboshi cultivars [46] leading the authors to conclude that these two protein isoforms were not important for determining grain hardness. Hin-B2 protein, particularly Hinb-2b, was reported by these authors as important contributors for grain hardness. Lines with the *Hinb-2b* alleles showed much higher average hardness index (HI) (59.7) than those with the *Hinb-2a* alleles (45.8) in F_2_ lines from the cross between Shikoku hadaka 84 (*Hina-a/Hinb-1b/Hinb-2b*; 79.2) and Shikoku hadaka 115 (*Hina-b/Hinb-1a/Hinb-2a*; 45.2) [46]. The MS peptide sequence data indicates that both Conrad and Lacey have *Hina-b/Hinb-1a/Hinb-2a* alleles. Hardness index calculated using the Single Kernel Characterization System (SKCS) analysis showed a significantly higher value for Conrad compared to Lacey (Table 3). The difference in the seed hardness values between the six-rowed Lacey and two-rowed Conrad was about 13 units, similar to the difference reported in the F_2_ lines [46]. Based on these contradictory data we speculate that developing protein markers (as opposed to DNA markers) for hordoindolines may provide a more reliable screen for the grain hardness trait in barley.

**Table 3:** Kernel hardness and other grain parameters of barley cultivars as determined by Single Kernel Characterization System

	Conrad	Lacey
Hardness	66.5	53.5
Diameter (mm)	2.6	2.5
Weight (mg)	31.4	26.0
Moisture (%)	11.9	10.8

## Conclusions

In this study a deep proteome analysis of barley seeds was undertaken using shotgun nano HPLC MS/MS. More than 900 of the 1168 proteins identified were annotated as ‘uncharacterized proteins’ or ‘predicted proteins’, suggesting that curation of barley genes needs a significant improvement. Identifying the orthologous proteins from the well-curated rice genome aided in conducting GO enrichment analysis. The comparative proteomics analysis between the six-rowed and two-rowed barley cultivars indicated only 20 proteins were differentially abundant between the two cultivars. Variation in the abundances of hordoindoline proteins was one of the key differences between the two-rowed Conrad and six-rowed Lacey. The type of hordoindoline proteins may contribute to the differences between the seed hardness of these two cultivars. This suggests that differences in protein profiles can provide a useful tool for examining more complex traits such as malting quality. Efforts are underway toward using this technique during various stages of malt production for identifying novel protein markers for predicting barley malting quality.

## Methods

Seeds of barley cultivars, Conrad (two-row) and Lacey (six-row) growing in Wyoming under irrigated conditions, collected from the 2014 field harvest were used for this study.

### Total protein extraction

Approximately 1 g of barley seeds (12–15 seeds) were sterilized with 70% ethanol for 10 s and then washed three times with distilled water. Sterilized seeds were frozen in liquid nitrogen in a pre-cooled mortar and ground to a fine powder with pestle. Approximately 100 mg of the finely ground powder was added to a pre-weighed 2 mL tube containing 1 mL of petroleum ether. The tubes were placed on a rotator at a gentle setting to ensure thorough mixing for 15 min. Samples were centrifuged for 5 min and the supernatant was decanted. The defatting process was repeated two more times. The pellets were air-dried and the proteins were extracted in a 1 mL solution containing 50 mmol L^−1^ Tris–HCl pH 8.8, 1.5 mmol L^−1^ KCl, 0.07% β-mercaptoethanol (β-ME), 1% Protease inhibitor cocktail (Promega) and 1% (*w/v*) SDS. Samples were placed on ice for 1 h with vortexing for 1 min every 15 min during this incubation step. The tubes were centrifuged for 15 min at 4^o^ C at 11,000 *g*. The supernatant was transferred to a pre-weighed centrifuge tube (Oak Ridge style, Nalgene). Four volumes of ice-cold acetone containing 0.07% β-ME was added, mixed thoroughly and incubated at -20^o^ C overnight. The precipitated proteins were collected by centrifugation at 18,400 *g* at 4^o^ C for 15 min. The pellet was washed with 1 mL of acetone containing 0.07% β-ME. The supernatant was discarded and the wash steps were repeated two more times. The pellet was air-dried for 10 min and the weight of the tube with the dry pellet was recorded. The protein pellet was solubilized in a urea buffer pH 8.5 (8 mol L^−1^ urea in 50 mmol L^−1^ NH_4_HCO_3_) using 100 μL of buffer/mg weight of pellet.

### Enzymatic “In Liquid” digestion

Extracted seed protein (200 μg) was TCA/acetone precipitated (9% TCA, 28% acetone final concentration) and the pellet re-solubilized and denatured in 30 μL of 8 M urea / 50 mM NH_4_HCO_3_ (pH 8.5) / 1 mM Tris–HCl for 5 min. Subsequently, this was diluted to 120 μl for reduction with 5 μL of 25 mM dithiotrietol, 10 μL of MeOH and 75 μL of 25 mM NH_4_HCO_3_ (pH 8.5). The tubes were incubated at 52^o^ C for 15 min and cooled on ice to room temperature. This was followed by addition of 6 μL of 55 mM iodoacetamide for alkylation and incubated in darkness at room temperature for 15 min. In the final step, 16 μL of 25 mM DTT were added to quench the reactions. Subsequently, 30 μL of trypsin/LysC solution (100 ng/μL trypsin/LysC Mix from Promega in 25 mM NH_4_HCO_3_) and 28 μL of 25 mM NH_4_HCO_3_ (pH 8.5) was added to 200 μL final volume. Digestion was conducted for 2 h at 42 ° C then an additional 15 μL of trypsin/LysC solution added (final enzyme:substrate ratio of 1:44) and digestion proceeded overnight at 37 ° C. The reaction was terminated by acidification with 2.5% TFA (Trifluoroacetic Acid) to 0.3% final concentration. Fifty micrograms of digested proteins (1/4^th^ digestion volume) were cleaned up using OMIX C18 SPE cartridges (Agilent, Palo Alto, CA) per manufacturer protocol and eluted in 20 μL of 60/40/0.1% ACN/H_2_O/TFA, dried to completion in the speed-vac and finally reconstituted in 50 μL of 0.1% formic acid.

### NanoLC-MS/MS

Peptides were analyzed by nanoLC-MS/MS using the Agilent 1100 nanoflow system (Agilent) connected to a new generation hybrid linear ion trap-orbitrap mass spectrometer (LTQ-Orbitrap Elite™, Thermo Fisher Scientific) equipped with an EASY-Spray™ electrospray source. Chromatography of peptides prior to mass spectral analysis was accomplished using a capillary emitter column (PepMap® C18, 3 μM, 100 Å, 150 × 0.075 mm, Thermo Fisher Scientific) onto which 1 μL of extracted peptides was automatically loaded. The nanoHPLC system delivered solvents A: 0.1% (*v/v*) formic acid, and B: 99.9% (*v/v*) acetonitrile, 0.1% (*v/v*) formic acid. Peptides were loaded at 0.50 μL/min over a 30-min period and eluted at 0.3 μL/min directly into the nano-electrospray with gradual gradient from 3% (*v/v*) B to 20% (*v/v*) B over 154 min. The elution process concluded with 12-min fast gradient from 20% (*v/v*) B to 50% (*v/v*) B at which time a 5-min flash-out from 50–95% (*v/v*) B took place. As peptides eluted from the HPLC-column/electrospray source, survey MS scans were acquired in the Orbitrap with a resolution of 120,000 followed by MS2 fragmentation of 20 most intense peptides detected in the MS1 scan from 300 to 2000 m/z. Redundancy was limited by dynamic exclusion.

### MS data analysis

Raw MS/MS data were converted to Mascot generic format (mgf) files using MSConvert (ProteoWizard: Open Source Software for Rapid Proteomics Tools Development). Resulting mgf files were used to search against Uniprot’s Barley (*Hordeum vulgare*) database with decoy reverse entries (124,660 total entries) using in-house Mascot search engine 2.2.07 (Matrix Science) with fixed carbamidomethylation on Cysteine, plus variable Methionine oxidation and Asparagine/Glutamine deamidation. Peptide mass tolerance was set at 15 ppm and fragment mass at 0.6 Da. Protein annotations, significance of identification and spectral based quantification was done with the help of Scaffold software (version 4.4.1, Proteome Software Inc., Portland, OR). Protein identifications were accepted if they could be established at greater than 99.0% probability within 1% False Discovery Rate (FDR) and contained at least two identified peptides. Protein probabilities were assigned by the Protein Prophet algorithm [47]. Proteins that contained similar peptides and could not be differentiated based on MS/MS analysis alone were grouped to satisfy the principles of parsimony.

Scaffold’s spectral counting strategy was employed to compare protein abundances between Conrad and Lacey seed samples. Data was normalized based on the total spectrum count of all the proteins in the most abundant sample. The Fisher’s Exact Test was used to compare the abundance of proteins based on spectral counts between Conrad and Lacey samples. This test was deemed more appropriate than the T-test because it directly calculates the probability of detecting the observed differences between the two samples, rather than relying on a large sample approximation. For this dataset a *p*-value of <0.00089 was considered statistically significant. Scaffold calculates the Fisher’s exact test *p*-value according to a model discussed earlier [48].

### Annotations and Gene Ontology analysis

Barley protein sequences were mapped back to Uniref90 and Uniref50 databases to obtain more functional information. Gene ontologies (GOs) for the categories of biological process, molecular function and cellular compartment were obtained through the Uniref database. CateGOrizer tool was used for identifying the major GO categories and generating a pie chart (http://www.animalgenome.org/cgi-bin/util/gotreei).

### Gene Ontology enrichment analysis

Barley protein sequences were used for batch BLAST analysis to identify the best matching rice homologs. The Uniprot identifiers were then used to identify the corresponding TIGR loci using Biomart tool in the Phytozome database (https://phytozome.jgi.doe.gov/biomart/martview), Rice ID checker tool in the Oryzabase (http://shigen.nig.ac.jp/rice/oryzabase/tool/riceIdChecker/search) and the Rice Pseudomolecule Version Converter tool in the MSU rice database (http://rice.plantbiology.msu.edu/analyses_search_converter.shtml).

Singular Enrichment Analysis tool in GO analysis toolkit and database for agricultural community, AgriGO (http://bioinfo.cau.edu.cn/agriGO/) was used to identify the GO terms enriched in the seed proteome.

### Hydropathy profile

Protein sequences of the barley seed proteome in FASTA format were obtained from the Uniprot database. The grand average of hydropathic value (GRAVY) was calculated using the gravy calculator (http://www.gravy-calculator.de/). The hydropathy plot was generated using MS Excel.

### Seed hardness test

Seeds of Conrad and Lacey cultivars were dehulled at the USDA Cereal Crops Research Unit malting lab (Madison, WI). About 300 dehulled seeds of Lacey and Conrad were then processed through the Single Kernel Characterization System (SKCS) instrument at the USDA Soft Wheat Quality Lab (Wooster, OH) to determine seed hardness.

## Additional files

Additional file 1: Figure S1. Two-row and six-row barley head. (PDF 2451 kb)
Additional file 2: Table S1. Transmembrane proteins in the barley seed proteome. (XLSX 40 kb)
Additional file 3: Table S2. List of the barley seed proteins identified from Conrad and Lacey cultivars. (XLSX 225 kb)
Additional file 4: Table S3. List of the enriched Gene Ontology terms in the barley seed proteome. (XLSX 39 kb)
Additional file 5: Figure S2. Gene Ontology enrichment analysis for molecular function category using AgriGO. (PDF 689 kb)
Additional file 6: Figure S3. Gene Ontology enrichment analysis for cellular compartment category using AgriGO. (PDF 428 kb)
Additional file 7: Table S4. List of the barley seed proteins with Uniprot identifiers, peptide counts, sequence coverage, LFQ intensities, MS/MS count. (XLSX 882 kb)
Additional file 8: Table S5. List of the barley seed proteins with descriptions derived from Uniprot, unique peptide count, spectrum count and percent protein coverage. (XLSX 355 kb)
Additional file 9: Figure S4. One-dimensional SDS PAGE analysis of the barley seed proteins from two-row Conrad and six-row Lacey cultivars. Twenty micrograms of the protein from each of the three replicates were loaded on a 10%SDS-PAGE. Gel was stained with Coomassie Brilliant Blue overnight. Following destaining, the gel was dried and photographed. (PDF 390 kb)

## Abbreviations

GO: Gene Ontology
GRAVY: Grand average of hydropathic value
HOR: Hordoindoline
