# Supplementary figures and images for: Shotgun proteomics of the barley seed proteome
Source: BMC Genomics. 2017 Jan 6;18:44. doi: 10.1186/s12864-016-3408-5 (PMC5219712; doi:10.1186/s12864-016-3408-5)

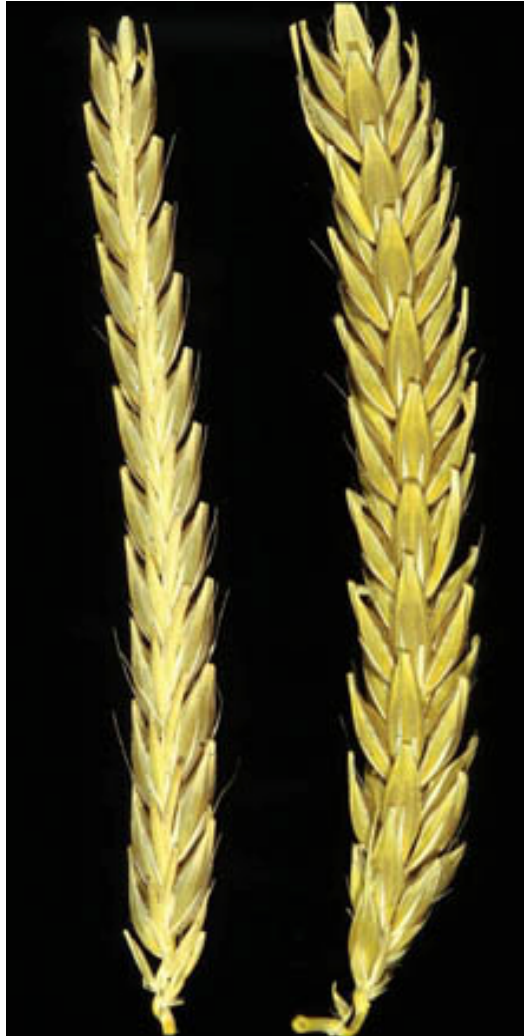

Two-rowed  
Conrad

Six-rowed  
Lacey

Supplement: Additional file 1: Figure S1. — Two-row and six-row barley head. (PDF 2451 kb) [file 12864_2016_3408_MOESM1_ESM.pdf]

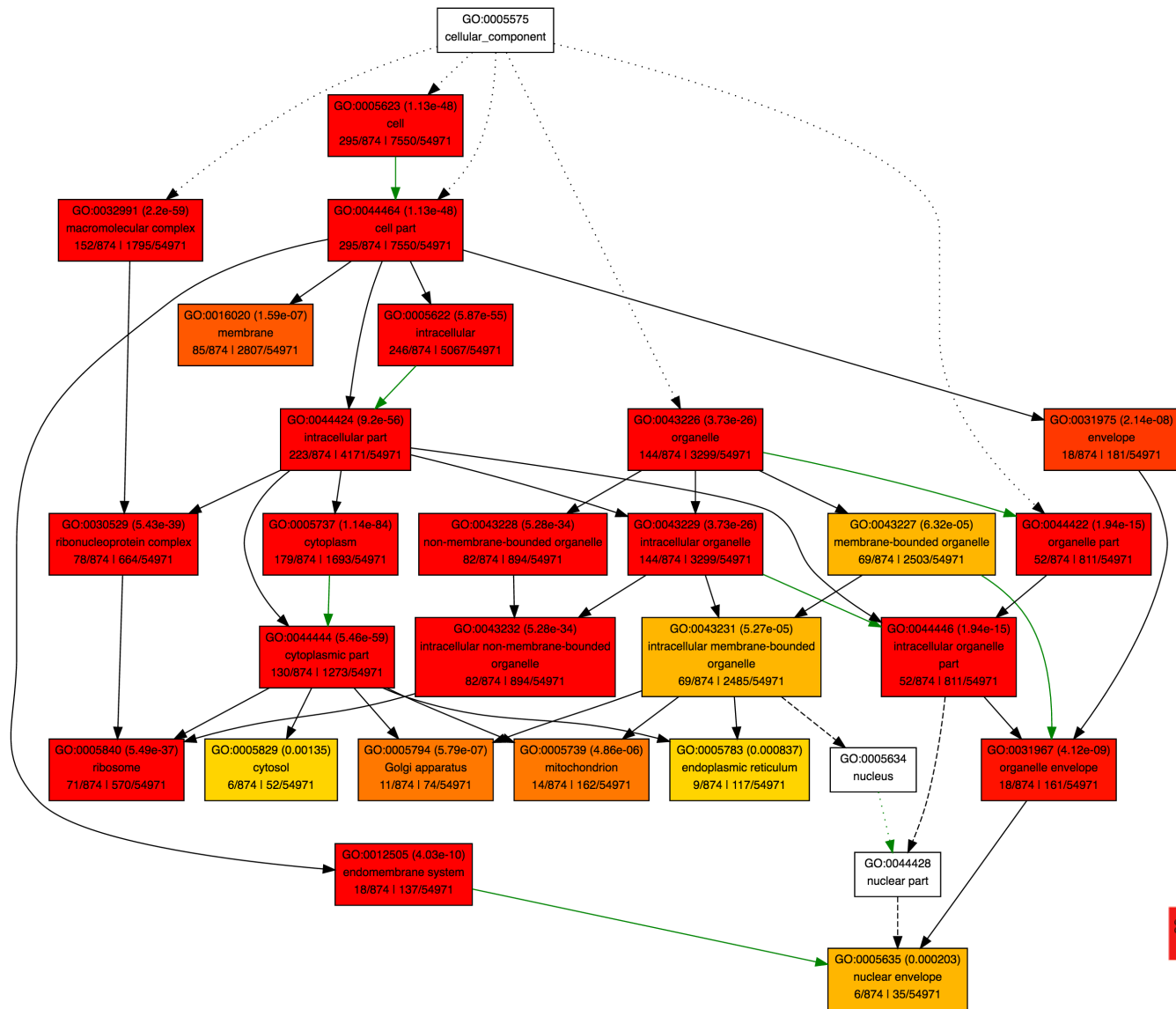

Supplement: Additional file 5: Figure S2. — Gene Ontology enrichment analysis for molecular function category using AgriGO. (PDF 689 kb) [file 12864_2016_3408_MOESM5_ESM.pdf]

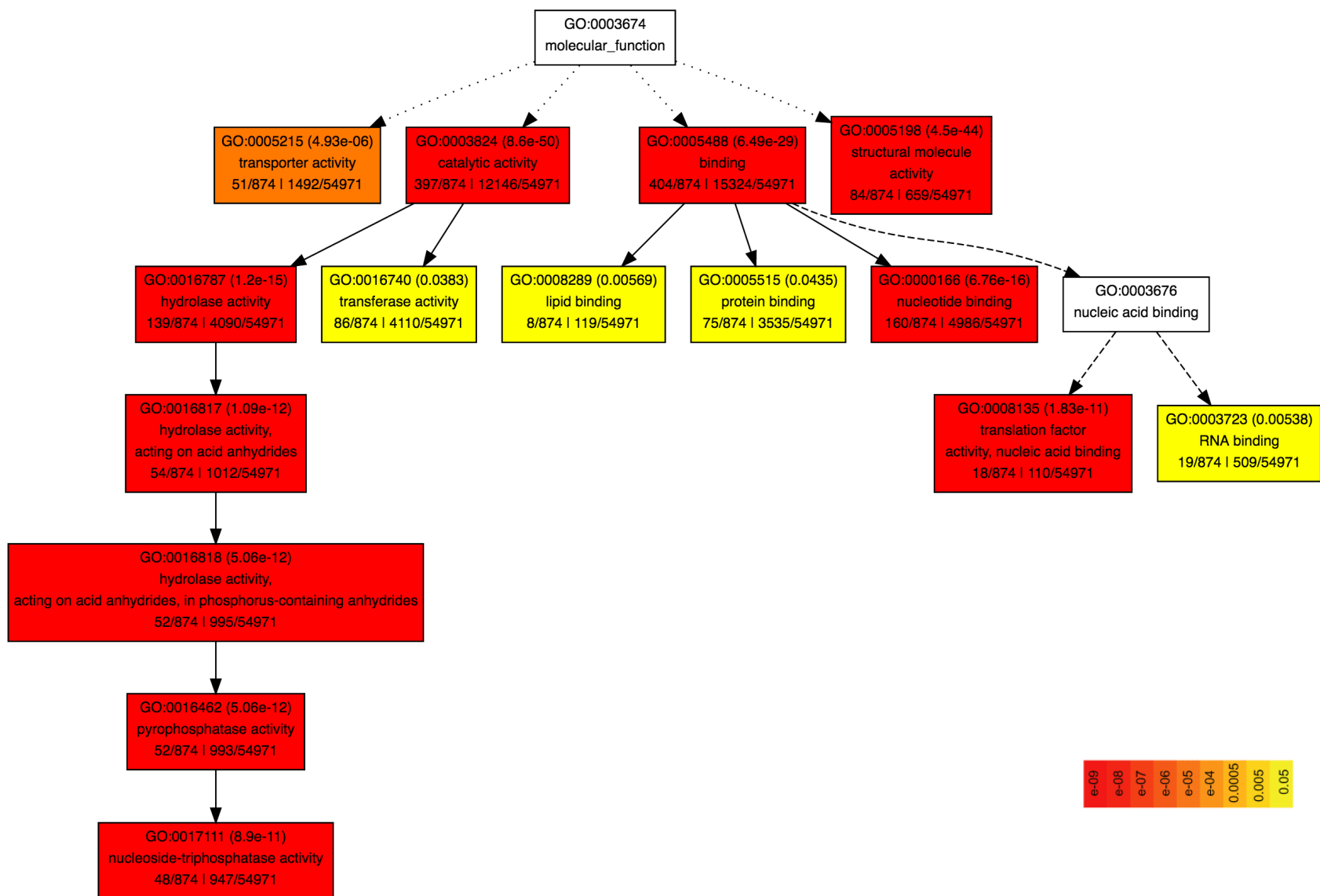

Supplement: Additional file 6: Figure S3. — Gene Ontology enrichment analysis for cellular compartment category using AgriGO. (PDF 428 kb) [file 12864_2016_3408_MOESM6_ESM.pdf]

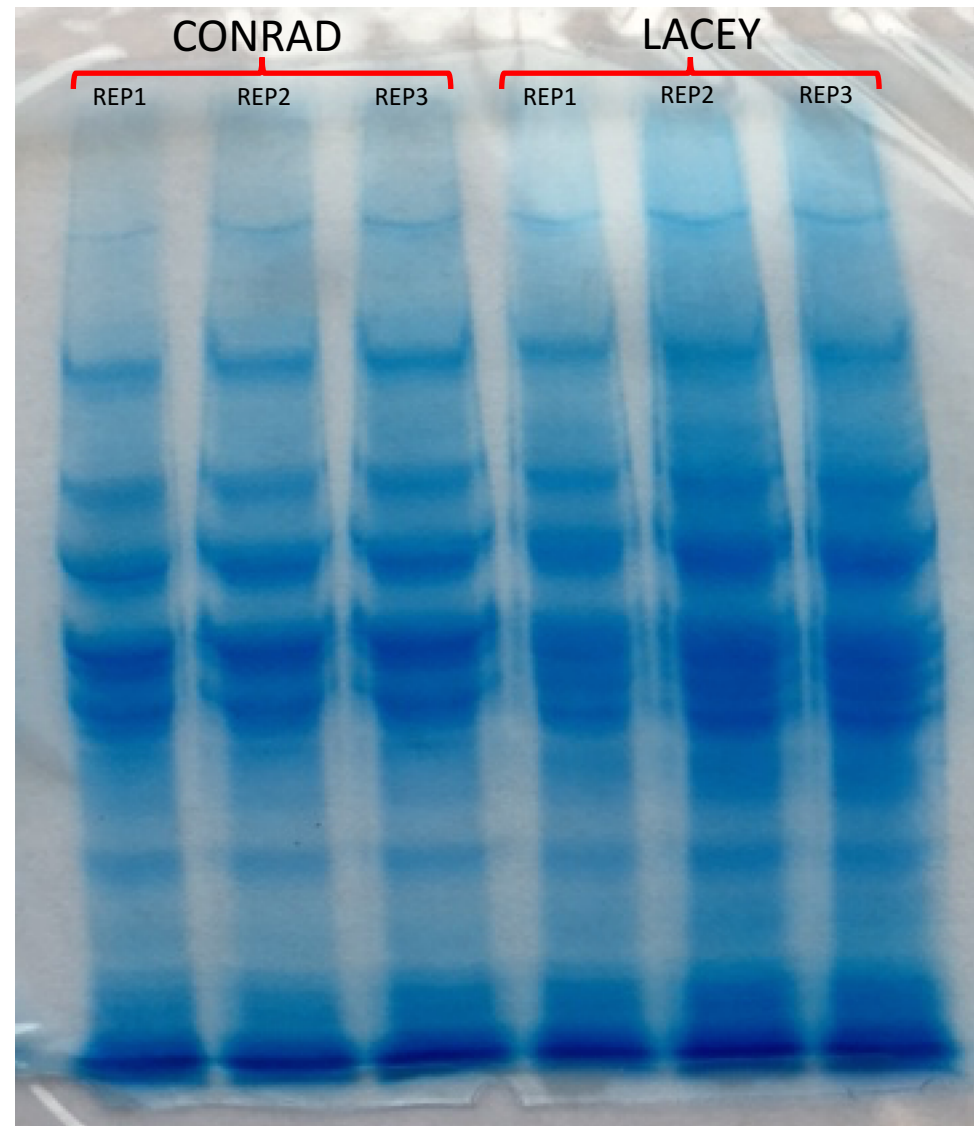

Supplement: Additional file 9: Figure S4. — One-dimensional SDS PAGE analysis of the barley seed proteins from two-row Conrad and six-row Lacey cultivars. Twenty micrograms of the protein from each of the three replicates were loaded on a 10%SDS-PAGE. Gel was stained with Coomassie Brilliant Blue overnight. Following destaining, the gel was dried and photographed. (PDF 390 kb) [file 12864_2016_3408_MOESM9_ESM.pdf]
